# Experience with microarray-based comparative genomic hybridization for prenatal diagnosis in over 5000 pregnancies

**DOI:** 10.1002/pd.3945

**Published:** 2012-08-02

**Authors:** Lisa G Shaffer, Mindy P Dabell, Allan J Fisher, Justine Coppinger, Anne M Bandholz, Jay W Ellison, J Britt Ravnan, Beth S Torchia, Blake C Ballif, Jill A Rosenfeld

**Affiliations:** 1 Signature Genomic Laboratories, PerkinElmer, Inc.Spokane, WA, USA; 2 Commonwealth Perinatal ServicesRichmond, VA USA

## Abstract

**Objective:**

To demonstrate the usefulness of microarray testing in prenatal diagnosis based on our laboratory experience.

**Methods:**

Prenatal samples received from 2004 to 2011 for a variety of indications (*n* = 5003) were tested using comparative genomic hybridization-based microarrays targeted to known chromosomal syndromes with later versions of the microarrays providing backbone coverage of the entire genome.

**Results:**

The overall detection rate of clinically significant copy number alterations (CNAs) among unbiased, nondemise cases was 5.3%. Detection rates were 6.5% and 8.2% for cases referred with abnormal ultrasounds and fetal demise, respectively. The overall rate of findings with unclear clinical significance was 4.2% but would reduce to 0.39% if only *de novo* CNAs were considered. In cases with known chromosomal rearrangements in the fetus or parent, 41.1% showed CNAs related to the rearrangements, whereas 1.3% showed clinically significant CNAs unrelated to the karyotype. Finally, 71% of the clinically significant CNAs found by microarray were below the resolution of conventional karyotyping of fetal chromosomes.

**Conclusions:**

Microarray analysis has advantages over conventional cytogenetics, including the ability to more precisely characterize CNAs associated with abnormal karyotypes. Moreover, a significant proportion of cases studied by array will show a clinically significant CNA even with apparently normal karyotypes. © 2012 John Wiley & Sons, Ltd.

## INTRODUCTION

The original goal of prenatal testing was the identification of trisomy 21, especially in women of advanced maternal age. Conventional cytogenetic analysis allows for the identification of not only aneuploidies, such as trisomy 21, but other numerical and structural aberrations of the chromosomes. With the examination of banded metaphase chromosomes through a light microscope, the resolution at which most structural changes of the chromosomes can be visualized reliably is a loss (deletion) or gain (duplication) of about 10 million base pairs (Mb) of DNA.

Microarray analysis is capable of detecting large chromosome imbalances identified by karyotyping and alterations much smaller than 10 Mb in size; several prospective studies have demonstrated the usefulness of microarrays in prenatal testing for detecting such alterations^1^–^15^ However, the detection rates of clinically significant copy number alterations (CNAs) varied among the studies mainly because of two factors: (1) the arrays used had differing formats, designs, probe densities, and resolutions and (2) the clinical indications included in the studies varied; some included all prenatal cases, whereas others included only cases with abnormal ultrasound findings. One meta-analysis of published data found that microarray analysis in prenatal diagnosis detected clinically significant genomic alterations in an additional 2.5% of cases over conventional cytogenetic analysis.^16^ When only cases with ultrasound anomalies were included in the meta-analysis, the detection rate of CNAs above that of karyotyping was 5.2%,^16^ which also included results of unclear clinical significance. The meta-analysis was also limited by the variety of arrays used, combination of multiple indications for study, and the relatively low number of prenatal cases (*n* = 751).^16^

Our current study represents the largest report of prospective prenatal cases tested by microarray analysis to date. The purpose is to demonstrate the usefulness of microarray testing compared with karyotyping for a variety of clinical indications in prenatal diagnosis. We show that microarray analysis has several advantages over conventional karyotyping, providing an increase in detection of clinically relevant alterations in 5% to 8% of cases (depending on the indication for study), the majority of which would not be detected by karyotype analysis.

## METHODS

Prenatal samples from amniotic fluid, chorionic villi, fetal blood, or products of conception were received by our laboratory from July 2004 through December 2011 for cytogenetic diagnosis using various microarrays targeted to known chromosomal syndromes with later versions of the microarrays providing backbone coverage of the entire genome (http://www.signaturegenomics.com/detection_rates.html). Although this laboratory participated in the National Institute of Child Health and Human Development-sponsored clinical trial in prenatal microarray testing,^17^ none of the samples reported here were received as a part of that study. All data used in the analyses presented here were gathered or generated during the process of clinically approved microarray-based comparative genomic hybridization testing for routine patient care. Excluding samples that failed to generate results, a total of 5003 samples were tested for a variety of indications.

Microarray analysis was performed as previously described.^4^ Results were reported to physicians as normal (no clinically significant CNA, with or without benign CNAs identified), with a variant of uncertain significance (VOUS), or clinically significant (abnormal). A VOUS is defined as an alteration of unclear clinical relevance that has not been previously identified in a laboratory's patient population, has not been reported in the medical literature, has not been found in publicly available databases, or does not contain any known disease-causing genes.^15^ All data were retained in our laboratory information management system. Data from all cases resulting in abnormal or VOUS results were also captured in our database [Genoglyphix® Chromosomal Aberration Database, Signature Genomics, Spokane, WA, USA]. For this study, our laboratory information management system and the Genoglyphix Chromosomal Aberration Database were searched to identify all prenatal cases. Each case was reviewed (by authors MPD and JAR) and categorized according to the result (i.e. normal, VOUS, or abnormal) and indication for study (IFS). Although cases may have had more than one IFS, cases were counted only once and placed into the category with the most significant risk for a chromosome abnormality. The stratification of categories was a known abnormal karyotype in which the family desired further characterization of the anomaly (*n* = 648), family history of a parent known to carry a chromosome rearrangement or imbalance (*n* = 62), fetal demise (*n* = 417), abnormal ultrasound (*n* = 2858), abnormal first or second trimester screen (*n* = 77), other family history of a genetic condition (*n* = 487), advanced maternal age (AMA) (*n* = 346), parental anxiety (*n* = 95), and other/not specified indications (*n* = 13). Terminated pregnancies were not considered to be fetal demises. Those cases referred for abnormal ultrasound findings were further stratified according to the clinical phenotype, including anomalies in multiple organ systems (*n* = 808), anomalies in single organ systems (*n* = 1773), isolated soft marker(s) (*n* = 77), other nonstructural anomalies (*n* = 134), and other/not specified (*n* = 66) (see Shaffer *et al*., accompanying article).2012 Cases with unclear results were further reviewed (by authors LGS and JAR) and in some cases, reassigned to the normal or abnormal groups as appropriate based on new knowledge gained from the medical literature and from our own experience since the initial reporting of the case. A subset of the 5003 cases have been previously published (*n* = 1878)^2^,^4^,^5^; therefore, some classifications have been updated since previous analyses. Detection rates for abnormal and VOUS results were calculated after removing cases referred with known abnormal karyotypes, family history of a parental rearrangement, and fetal demise because of the increased chance (bias) of detecting clinically significant CNAs in these samples. Abnormal results were further stratified based on the size of the alteration (by author JAR). If the abnormality was an unbalanced translocation, the largest chromosomal segment affected by the translocation determined whether the case was placed in the ≥10 Mb or <10 Mb category.

## RESULTS

Cases were received from clinicians and laboratories from the US and abroad. Specimen types tested include cultured amniocytes (*n* = 3269, 65%), direct amniotic fluid (*n* = 343, 7%), cultured chorionic villi (*n* = 854, 17%), direct chorionic villi (*n* = 63, 1%), fetal blood (*n* = 25, 0.5%), products of conception (*n* = 432, 9%), and DNA from unspecified sources (*n* = 17, 0.3%). For ongoing pregnancies from November 2007 to December 2011, the average (mean) turnaround time between time of specimen receipt and initial reporting of array results was 7.5 days (range: 1–75 days, median 6 days); common reasons for delays included time for culturing, insurance verification, and holding the specimen for other test results.

After excluding potentially biased cases, the overall detection rate of clinically significant results was 5.3% (207/3876). The data were further stratified based on the IFS, and these detection rates and rates of VOUS are shown in Table 1. As predicted, fetal demises had a significantly higher detection rate of clinically significant results (8.2%, 34/417) than the rest of the IFS combined (5.3%, 207/3876) (two-tailed *p* = 0.024, Fisher's Exact test).

**Table 1:** Detection rates of abnormal and unclear array results, excluding cases referred with known abnormal karyotypes and family history of a rearrangement carrier parent

Indication	Normal (%)	Unclear (%)	Significant (%)	Total
Abnormal ultrasound	2462 (88.5)	135 (4.9)	184 (6.6)	2781
Abnormal ultrasound: only soft markers^4^	72 (93.5)	3 (3.9)	2 (2.6)	77
Abnormal MSS	68 (88.3)	5 (6.5)	4 (5.2)	77
Family history^5^	461 (94.7)	11 (2.3)	15 (3.1)	487
AMA alone	337 (97.4)	8 (2.3)	1 (0.3)	346
Anxiety alone	94 (98.9)	1 (1.1)	0 (0.0)	95
Other or not specified	12 (92.3)	0 (0.0)	1 (7.7)	13
**Total (nondemise)**	**3506 (90.5)**	**163 (4.2)**	**207 (5.3)**	**3876**
Fetal demise	359 (86.1)	24 (5.8)	34 (8.2)	417

AMA, advanced maternal age; MSS, maternal serum screening.

Soft markers include choroid plexus cysts, echogenic foci in the heart or bowel, isolated short long bones, absent nasal bone, single umbilical artery, persistent umbilical vein, wide gap between first and second toes, and fifth finger clinodactyly.

These family history referrals include *de novo* chromosome rearrangements in a previous child or other relative of the parents, genetic conditions in family members not caused by of chromosome abnormalities, and family members with conditions, such as intellectual disability or autism, of unknown or undiagnosed causes.

Of the 5003 prenatal specimens, 56.3% (*n* = 2819) were referred with normal karyotypes, 13% (*n* = 648) had known abnormal fetal karyotypes at the time of array testing, 16% (*n* = 802) had karyotyping carried out concurrently with microarray testing, and 14.7% (*n* = 734) had an unknown status of karyotypes. Excluding the biased family history and fetal demise categories, the detection rate of clinically significant CNAs was 5.5% (140/2533) among cases with known normal karyotypes, demonstrating that, in general, these detection rates from the whole cohort in this study represent the identification of clinically significant CNAs beyond those detected by karyotype analysis.

**Figure 1:**
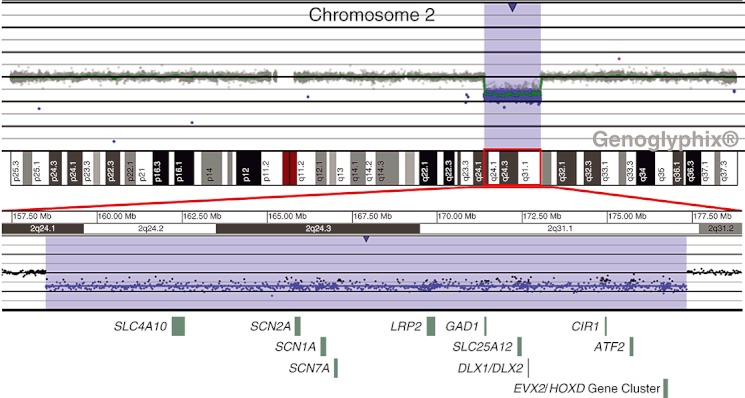
An 18.9-Mb 2q24q31 deletion identified by microarray in a specimen with a 46, XY karyotype. Microarray plots show a single-copy loss of 806 oligonucleotide probes from the long arm of chromosome 2, identified in cultured amniocytes from a fetus referred with a diaphragmatic hernia, Dandy Walker malformation, ventricular septal defect, and lower limb abnormalities. The deletion includes several disease-associated genes (green boxes), including epilepsy-associated *SCN1A* and *SCN2A* and limb abnormality-associated *HOXD* genes. Probes are ordered on the *x*-axis according to physical mapping positions (hg18), with the most distal 2p probes to the left and the most distal 2q probes to the right. Values along the *y*-axis represent log_2_ ratios of patient:control signal intensities. Results are visualized using Genoglyphix (Signature Genomics)

Table 2 shows the stratification of the clinically significant results, from Table 1, into the <10 Mb and ≥10 Mb categories, representing the reliable resolution for traditional karyotyping. Overall, 71% of the abnormalities identified were <10 Mb and not expected to be identified by conventional cytogenetic analysis. Among these, known microdeletion syndromes were detected in 35 cases (Table 3). In addition, 48% (33/69) of cases with abnormalities detected by microarray ≥10 Mb were reported with normal conventional karyotypes from the referral laboratories; 6% (4/69) had revised, abnormal karyotypes following the receipt of array results. Figure 1 shows an example of a large, ∼19-Mb CNA that was missed by routine cytogenetic testing. In 12 cases, disagreement between mosaic array results and karyotype may have been due to confined placental mosaicism, previously undetected mosaicism, or cultural artifact (especially when the specimen had been in long-term culture before array testing). Three cases were most likely artifact and were not classified as abnormal for this study and are not counted in the 33 cases above, while 9 cases did not have sufficient evidence to rule out cryptic mosaicism (Table 4).

**Table 2:** Data on clinically significant CNAs found in prenatal and fetal demise referrals, excluding those with abnormal karyotypes known at the time of testing and family history of a rearrangement carrier parent, stratified according to the size of the alteration identified

Abnormality	<10 Mb	≥10 Mb
Known microdeletion syndrome	35	NA
Known microduplication syndrome	3	NA
Microdeletion: reduced penetrance	46	NA
Microduplication: reduced penetrance	16	NA
Homozygous deletion	2	0
Terminal deletion	8	1
Terminal duplication	0	1
Other interstitial deletion	27	10
Other interstitial duplication	10	1
Unbalanced translocation	7	14
Insertion	2	0
Autosomal aneuploidy	NA	11
Sex chromosome aneuploidy	NA	6
XX male	NA	1
Polyploidy	NA	1
Complex rearrangements	12	7
Mosaic findings	4	16
Total	172 (71%)	69 (29%)

CNAs, copy number alterations; NA, not applicable.

**Table 3:** Summary of microdeletions, microduplications, and terminal abnormalities seen in on-going pregnancies and fetal demise referrals, excluding those with known abnormal karyotypes and family history of a rearrangement carrier parent

Microarray finding	Number of deletions	Number of duplications
**Known microdeletion or microduplication syndrome**		
Beckwith–Wiedemann syndrome, 11p15 deletion	1	NR
12q14q15 microdeletion syndrome	1	NR
PW/AS 15q11q13 deletion	2	Reduced penetrance: 1
Rubenstein–Taybi Syndrome (*CREBBP* 16p13.3 deletion)	1	NR
Alveolar Capillary Dysplasia with Misalignment of Pulmonary Veins (*FOXF1* 16q24.1 deletion)	1	NR
HNPP (17p12)	5	0
17q23.2 microdeletion	1	0
22q11.21 deletion syndrome	12^8^	Reduced penetrance: 5^8^
2p15p16 microdeletion syndrome	1	NR
Capillary malformation-arteriovenous malformation (*RASA1* 5q14.3 deletion)	1	NR
Congenital contractural arachnodactyly/Beals syndrome (*FBN2* 5q23.3 deletion)	1	NR
Greig cephalopolysyndactyly syndrome (*GLI3* 7p14.1 deletion)	1	NR
8p23.1 microdeletion/microduplication	1	1
9q34.3 microdeletion (*EHMT1* deletion)	1	NR
*DMD* deletion	2 males^8^, 2 carrier females	NR
*SHOX* deletion	2^8^	NR
*STS* deletion	3 males, 4 carrier females	Benign
14q22q23 microdeletion syndrome	1	NR
Simpson-Golabi-Behmel syndrome (*GPC3* Xq26.2 deletion)	1	NR
Split hand/foot (*FBXW4* 10q24 duplication)	NR	1
7q11.23 microduplication (reciprocal to WBS)	0	1
**Microdeletions and microduplications with reduced penetrance**		
15q11.2 BP1-BP2 microdeletion	19^8^	Benign
Distal 16p11.2 microdeletion/microduplication	3	3^8^
Proximal 16p11.2 microdeletion/microduplication	3	2^8^
16p12.1 microdeletion	3	Benign
16p13.11 microdeletion	4	Unclear: 9
17q12 microdeletion (RCAD)	4	0
1q21.1 proximal microdeletion (TAR)/microduplication	6	5
1q21.1 distal microdeletion/microduplication	3^8^	4
22q11.21 atypical microdeletion	1	Unclear: 3
22q11 distal microdeletion	1	0
*NRXN1* deletion	2	NR
**Terminal chromosome abnormalities**		
1p	1	1^9^
1q	1^9^	1^9^
4p	5^8^,^9^	0
4q	1^9^	4^8^,^9^
5p	1^9^	0
5q	1^9^	1^9^
6p	3^8^	0
6q	1^9^	2^8^
7p	0	4^8^,^9^
7q	6^8^,^9^	1^9^
8p	1^9^	2^9^
8q	0	1^9^
9p	1^8^	0
9q	2^9^	0
10p	0	1^9^
10q	1^9^	1^9^
11p	0	1^9^
13q	2^9^	0
14q	1	1^9^
16p	1^9^	1^9^
17p	3^9^	0
17q	0	2^8^,^9^
18p	1^8^	0
18q	1^9^	2^8^
19q	0	1^9^
20p	0	1^9^
20q	0	1^9^

HNPP, hereditary neuropathy with liability to pressure palsies; NR, nonreciprocal: deletions or duplications are not mediated by nonallelic homologous recombination, so recurrent reciprocal rearrangements are not reported here; PW/AS, Prader-Willi/Angelman syndrome; RCAD, renal cysts and diabetes; TAR, thrombocytopenia/absent radius; WBS, Williams–Beuren syndrome.

Includes case(s) classified as complex; additional abnormalities were present.

Includes case(s) with unbalanced translocations.

**Table 4:** Mosaic abnormalities ≥10 Mb seen by microarray but not by karyotype

Microarray finding	FISH analysis	Sample type	Karyotype	Interpretation^11^
Mosaic trisomy 7	Trisomy 7 in 7/30 (23%)	Cultured villi	46,XX (cultured villi)	CPM or FM
Mosaic trisomy 16	Trisomy 16 in 2/150 (1%)	Cultured amniocytes	46,XX (cultured amniocytes)	Artifact or FM
Mosaic monosomy X	Monosomy X in 15/17 (88%)	Cultured amniocytes	46,XX (cultured amniocytes)	Artifact or FM
Mosaic supernumerary der(2)	Der(2) in 55/120 (46%)	Cultured amniocytes	46,XX (cultured amniocytes)	Likely FM
Mosaic 6-Mb 4q31 gain; mosaic 27-Mb 4q31q34 gain	Dup(4) in 10/60 (17%)	Cultured amniocytes	46,XX (cultured amniocytes)	Likely FM
Mosaic 12-Mb 16p11p12 loss	Del(16) in 13/55 (24%)	Cultured villi	46,XX (cultured villi)	Artifact or CPM
Mosaic trisomy 2	Not possible	Cultured villi (isolated from POC)	46,XX (cultured villi)	Artifact, CPM, or FM
Mosaic trisomy 8 & trisomy 20	Trisomy 8 in 13/30 (43%); trisomy 20 in 10/30 (33%)	Cultured villi	46,XX (cultured villi)	Artifact, CPM, or FM
Mosaic trisomy 22	Trisomy 22 in 11/30 (37%)	Cultured amniocytes	46,XY (cultured villi)	Artifact or FM
Mosaic 50-Mb 2q32q37 gain	Dup(2) in 8/30 (27%)	Cultured villi	46,XX (cultured villi)	Likely artifact
Mosaic monosomy X	Monosomy X in 37/50 (74%)	Cultured villi	46,XY (cultured villi)	Likely artifact
Mosaic trisomy 3	Tetraploidy with pentasomy 3 in 8/30 (27%); tetraploidy in 2/30 (7%)	Cultured amniocytes	46,XX (cultured amniocytes)	Likely artifact

FISH, fluorescence *in situ* hybridization; CPM, confined placental mosaicism; FM, fetal mosaicism; POC, products of conception.

Interpretations are the final interpretation based on all results from follow-up testing (when available), phenotypic information, and time in culture.

Detection rates for clinically significant abnormalities in cases with a known fetal chromosome abnormality or a family history of a parental rearrangement were calculated separately and are shown in Table 5. Combining all known abnormal karyotypes, 1.4% showed clinically significant CNAs elsewhere in the genome not associated with the known rearrangement.

**Table 5:** Detection rates for cases with a known fetal chromosome abnormality in an on-going pregnancy or fetal demise, or a family history of a parental rearrangement

Karyotypic abnormality	Imbalance related to the known karyotype (%)	No imbalance (%)	Other unrelated finding, significant (%)	Other unrelated finding, unclear (%)	Total
**Nonmosaic, apparently balanced rearrangement**	**19 (7.9)**	**207 (86.6)**	**4 (1.7)**	**9 (3.8)**	**239**
Balanced translocation	15 (7.9)	166 (87.8)	4 (2.1)	4 (2.1)	189
Inversion	2 (4.5)	37 (84.1)	0 (0.0)	5 (11.4)	44
Insertion	2 (33.3)	4 (66.7)	0 (0.0)	0 (0.0)	6
**Mosaic, apparently balanced rearrangement**	**0 (0.0)**	**5 (100.0)**	**0 (0.0)**	**0 (0.0)**	**5**
**Nonmosaic, apparently unbalanced rearrangement**	**183 (67.5)**	**82 (30.3)**	**3 (1.1)**	**3 (1.1)**	**271**
Marker or ring chromosome	47 (53.4)	38 (43.2)	1 (1.1)	2 (2.3)	88
Suspected deletion	59 (84.3)	10 (14.3)	0 (0.0)	1 (1.4)	70
Suspected duplication	34 (57.6)	25 (42.4)	0 (0.0)	0 (0.0)	59
Complex rearrangements	15 (68.2)	5 (22.7)	2 (9.1)	0 (0.0)	22
Aneuploidy	16 (100.0)	0 (0.0)	0 (0.0)	0 (0.0)	16
Unbalanced translocation	12 (75.0)	4 (25.0)	0 (0.0)	0 (0.0)	16
**Mosaic, apparently unbalanced rearrangement**	**44 (40.0)**	**62 (56.4)**	**2 (1.8)**	**2 (1.8)**	**110**
Marker or ring chromosome	41 (44.6)	47 (51.1)	2 (2.2)	2 (2.2)	92
Other mosaic unbalanced karyotype	3 (16.7)	15 (83.3)	0 (0.0)	0 (0.0)	18
**Variant**	**4 (28.6)**	**10 (71.4)**	**0 (0.0)**	**0 (0.0)**	**14**
**Mismatched genotypic and phenotypic sex**	**9 (100.0)**	**0 (0.0)**	**0 (0.0)**	**0 (0.0)**	**9**
**Total, abnormal karyotype**	**259 (40.0)**	**366 (56.5)**	**9 (1.4)**	**14 (2.2)**	**648**
**Family history of a chromosome rearrangement in a parent**	**33 (53.2)**	**27 (43.5)**	**0 (0.0)**	**2 (3.2)**	**62**

## DISCUSSION

With the advent of microarray-based comparative genomic hybridization in prenatal diagnosis, a substantially larger proportion of clinically relevant chromosomal abnormalities can be identified, with the exception of some triploidies,^19^ balanced rearrangements, and cases of uniparental disomy. The resolution of detectable alterations has improved from about 10 Mb or larger-sized rearrangements with karyotyping to a few kb in size with microarray analysis, thus increasing the number of identifiable abnormalities in prenatal diagnosis.

### Detection rates of microarray-based comparative genomic hybridization

Prenatal samples over a period of ∼7 years were examined for detection of CNAs by microarray analysis. With the exception of parental anxiety cases (*n* = 95), all indications for study had clinically significant alterations identifiable by microarray. As with other studies that used varying array platforms over time,^3^,^13^,^14^ the cases reported here were also tested with various array platforms and designs.^2^,^4^,^15^ Regardless, the overall detection rate of clinically significant CNAs among unbiased, nondemise prenatal samples for any IFS was 5.3% and was 6.5% when only pregnancies with abnormal ultrasound findings were considered. It is important to note that when we consider only cases tested on oligonucleotide-based arrays, the overall detection rate increases to 6.5% (182/2818), and in the subset with abnormal ultrasound findings it increases to 7.6% (165/2161). For all of our unbiased, nondemise prenatal samples, positive detection rates among the IFS categories ranged from 0.3% for AMA to 8.2% for fetal demise (Table 1). On the basis of the 3876 ‘unbiased’ prenatal array cases, for which known abnormal fetal karyotypes, family history of a chromosome abnormality, and fetal demise cases were removed, the rate of detecting significant abnormalities was dependent on the various broad IFS categories (χ^2^ = 38.03, *df* = 6, *p* < 0.0001). Thus, while significant CNAs can be found among most categories of IFS, they are more likely to be found in certain categories, demonstrating an increased diagnostic utility of microarray testing among certain subsets of prenatal patients.

Large prospective studies using microarrays for prenatal testing are necessary to assess the detection rates of chromosome abnormalities beyond those detected by karyotyping. In addition to the multicenter National Institute of Child Health and Human Development-sponsored clinical trial,^17^ five recent prospective prenatal studies have been published using relatively high-resolution arrays.^10^,^13^,^14^,^20^ Three of these studies used bacterial artificial chromosome-based arrays while two^13^,^14^ used bacterial artificial chromosome arrays and oligonucleotide arrays in later years. Combined, these studies tested over 10 000 pregnancies with microarrays and found detection rates over all IFS of ∼1% to ∼6%. Two studies^13^,^14^ found 9% to 11% clinically significant abnormal array results in cases with abnormal ultrasound findings.^13^,^14^ The results from these studies are comparable to our results of clinically significant CNAs in 5.3% for any IFS and 6.5% for pregnancies with abnormal ultrasound findings. In addition, we report an 8.2% detection rate in those cases referred for fetal demise or stillbirth. Specifically in these fetal demise or stillbirth cases, microarray analysis should be considered a superior alternative to karyotyping because DNA can be extracted directly from these hard-to-grow specimens without the need for culturing.

For cases with AMA as the only IFS, previous studies varied in their detection of CNAs that were not identified by karyotype. Fiorentino *et al*.2011 found one *de novo* 15q13.1q13.3 deletion (1/444, 0.2%); Armengol *et al*.2012 found two *de novo* microduplications (2/56, 3.6%); Breman *et al*.^13, personal communication^ found five abnormalities, three of which were undetectable by karyotype, including a deletion of 16p13.11 and two microduplications (3/394, 0.76%); and Lee *et al*.2012 found 30 abnormalities, ten of which were undetectable by karyotype, including duplication of 22q11.21, Williams–Beuren syndrome, *STS* deletion, and hereditary neuropathy with liability to pressure palsies (10/1911, 0.52%). These findings are comparable to our detection of one significant abnormality among AMA cases (1/346, 0.3%). This case had a *de novo* microduplication of distal 16p11.2, which contains *SH2B1*, has been reported in individuals with developmental delay and congenital anomalies,^21^ and has been shown to be enriched among individuals with abnormal phenotypes.^22^ It is interesting that the other studies also found recurrent microdeletions and microduplications in their AMA populations. Notably, Lee *et al*.2012 found a comparable rate of abnormalities in their non-AMA, parental anxiety group (5/989, 0.51%), suggesting that there may be a background risk of ∼0.5% in the general population for the identification of a significant microdeletion or microduplication. Combining our data with that of these four other studies,^11^,^13^,^14^,^20^ 0.5% of AMA (17/3151) had abnormalities detected by array that would not be detectable by karyotyping. Furthermore, assuming 200 000 invasive prenatal tests are performed annually in the United States for AMA, parental anxiety, and/or abnormal maternal serum/first trimester screening, a detection rate of at least 0.5% in this population means a significant number of pregnancies, at least 1000, would be identified with a clinically significant, submicroscopic CNA if tested by microarray.

### Ability of microarray to detect abnormalities missed by traditional cytogenetic analysis

**Figure 2:**
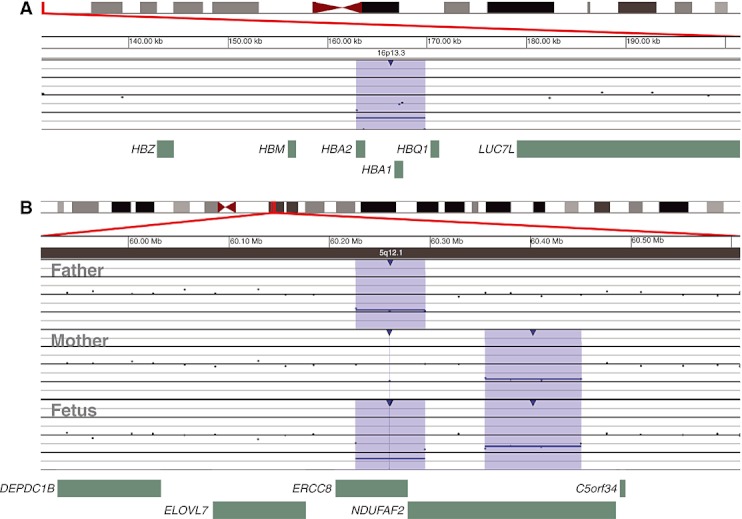
Homozygous deletions of recessive loci in prenatal specimens. (A) Microarray plot showing a complex pattern of heterozygous and homozygous deletion of 5 oligonucleotide probes from the short arm of chromosome 16 over the alpha thalassemia locus at 16p13.3, identified in cultured amniocytes from a fetus referred with edema. Probes are ordered on the *x*-axis according to physical mapping positions (hg18), with the most distal 16p13.3 probes to the left and the most proximal 16p13.3 probes to the right. Values along the *y*-axis represent log_2_ ratios of patient:control signal intensities. Parental samples were unavailable for array comparative genomic hybridization, but blood work was suggestive of both parents being carriers for alpha thalassemia. (B) Microarray plots from parents and fetus in a prenatal case referred for a family history of intellectual disability. After fetal testing, inspection of this region in the parents showed that the father has a single-copy loss of 3 probes, overlapping *ERCC8*, a gene associated with Cockayne syndrome, and *NDUFAF2*, a gene associated with mitochondrial complex I deficiency. The mother has a complex finding of a heterozygous deletion of a single probe within *ERCC8* and a more distal heterozygous deletion within *NDUFAF2*. The fetus inherited both deletions and is therefore predicted to have homozygous loss-of-function of *ERCC8* and *NDUFAF2*. Probes are arranged as in (A), with the most proximal 5q12.1 probes to the left and the most distal 5q12.1 probes to the right. Genes within the region are shown as green boxes. Results are visualized using Genoglyphix (Signature Genomics)

Of the clinically significant CNAs identified in this study, 71% were less than 10 Mb in size. Among these were 35 deletions of well-documented microdeletion syndromes such as DiGeorge and Prader-Willi/Angelman syndromes; 46 microdeletions associated with reduced penetrance; 3 cases of well-described microduplication syndromes; 16 microduplications associated with reduced penetrance; 8 terminal deletions, including monosomy 1p36 and deletion 7q; 7 cryptic, unbalanced translocations; and 12 complex rearrangements involving two or more chromosomal segments, not presenting as an unbalanced translocation after fluorescence *in situ* hybridization visualization (Tables 2 and 3). Because many of these abnormalities are below the resolution of karyotyping, they are unlikely to be detected by routine analysis of fetal chromosomes. Because clinically significant alterations were as small as 7 kb (Figure 2), these data also support that laboratories should not use an arbitrary reporting cut-off based on size as previously proposed^23^ because significant small aberrations will be missed. Alterations should be examined for genetic content and reported based on those findings.

### Interpretation of copy number alterations

The laboratory must interpret results into one of a minimum of four categories: normal (no alterations), normal with benign CNA(s), VOUS, or abnormal (clinically significant). In this study, cases with results reported as having unclear clinical significance were re-reviewed and in some cases reassigned as benign variants (about three-fourths of the reclassifications) or clinically significant CNAs (about one-fourth of the reclassifications). The majority of the cases reclassified as significant were microdeletions and microduplications of regions that are now well recognized as syndromes with a wide range of features because of variable penetrance and expressivity.^22^ These include deletions of distal 16p11.2, 15q11.2, and 16p12.1; atypical deletions in 22q11.21; and duplications of distal 16p11.2 and proximal and distal 1q21.1. Interestingly, many of these alterations were identified in a parent (21/34 with known inheritance).

With the increased ability to detect CNAs with microarrays, VOUS will be identified. In this study, 4.2% of nondemise cases remained unclear. Some laboratories discriminate between those VOUS that are inherited from a parent and those that are *de novo* in origin, placing more potential clinical relevance on those that are *de novo*, while grouping the majority of those inherited as benign,^11^,^13^,^14^,^20^ thus effectively lowering the rates of reported VOUS. Our laboratory has typically reported higher rates of VOUS^15^ because inheritance from a parent often does not help assign the clinical relevance of the alteration because of the possibility of incomplete penetrance or variable expressivity.^22^ This is especially true in the laboratory setting in which clinical information on parents is limited. Thus, reporting criteria seem to differ between laboratories regarding VOUS. In our study, 0.39% (*n* = 15) had apparently *de novo* VOUS, and 0.75% (*n* = 29) had VOUS of unknown inheritance. The sum of these (1.1%) is comparable to VOUS rates in other published studies.^13^,^14^,^20^

Finding a VOUS prenatally can create challenges in counseling expectant parents, and it has been recommended to provide such information in a complete and nondirective manner.^24^,^25^ Although microarray testing can introduce uncertainty (and associated anxiety) into a pregnancy by the reporting of a VOUS, this risk should be considered in comparison to the likelihood of identifying clinically significant findings when deciding whether to perform the testing: for example, 6.6% of structural ultrasound abnormality cases and 2.6% of cases with soft marker findings had abnormal results. Additionally, even in those cases in which a VOUS is identified, this result has excluded the presence of a few hundred chromosomal alterations that lead to well-characterized syndromes in the fetus.

For the most part, the detection rates in this study are in addition to the chromosome abnormalities found in routine cytogenetic testing because the cases reported here had normal karyotypes or chromosome analysis was in progress at the time of the microarray testing. Given this added ability to detect significant chromosome alterations in fetuses with abnormalities, this fully justifies the use of microarray testing in trying to identify the etiology of the clinical phenotypes, and thus microarray should be considered as the first test or used concurrently with conventional karyotyping.

### Identification of recessive disease risk prenatally

In 2011, our laboratory began reporting fetal cases identified as carriers of recessive disease. In the 1351 nondemise cases tested, 27 (2%) had a heterozygous deletion that included a recessive gene locus. In examining all cases, two had homozygous deletions that are predicted to result in disease, alpha thalassemia in one and Cockayne syndrome and mitochondrial complex I deficiency in the other (Figure 2). Recessive disease may result from homozygous deletion of a recessive disease locus or by heterozygous deletion of one allele and mutation in the other allele.^26^ There are now several documented examples of these phenomena,^26^,^27^ and fetuses with heterozygous deletions identified by microarray analysis could be tested further to exclude a mutation in the intact allele. Correlation of the phenotype, family history, and ethnic background with the particular disease identified can help guide further actions.

### Phenotypic implications of copy number alterations

Part of the difficulty in interpreting some clinically significant microarray results is that much of the information available about the clinical consequences of the CNAs may be biased toward increased severity because the CNAs were initially identified in children studied by microarray as a result of some phenotypic abnormality. This biased sampling does not allow for understanding the entire spectrum of the phenotype because clinically normal individuals are rarely investigated by microarrays. In these cases of rare or poorly understood CNAs, case-control studies can be used to understand whether the CNA is likely pathogenic.^22^,^28^ In addition, although ultrasound can detect fetal anomalies, it cannot detect conditions such as intellectual disability, developmental delay, autism, seizure disorders, and even conditions such as schizophrenia. Microarrays will uncover CNAs that can result in these conditions and identify at-risk fetuses, which is a benefit of the test but also could be a source of parental anxiety. Such issues surrounding phenotypic spectrums and incomplete penetrance should be part of patient pretest counseling for prenatal microarray testing.

### The use of microarrays with known abnormal fetal karyotypes

Finally, 634 on-going or terminated pregnancies and 14 cases of fetal demise were referred because of a known chromosome abnormality in the fetus of which the family desired further molecular characterization. In these cases, CNAs associated with the reported chromosomal rearrangement were identified in 40.0%; the array results, therefore, allowed more precise characterization of the size and genomic content of the chromosome anomaly. In contrast, 60.0% of rearrangements showed no gain or loss of the genomic region implicated by conventional chromosome analysis. Thus, in some cases, apparently balanced translocations were balanced (no gain or loss of DNA at the breakpoints based on the resolution of the array used), marker chromosomes did not contain any detec table euchromatin, or other suspected imbalances did not affect euchromatin (Table 5). It is interesting to consider in this context that if microarrays were a first-tier test in prenatal diagnosis, only unbalanced karyotypes would be identified, and those cases that are truly balanced would not be discovered. However, because the discovery of balanced translocations and understanding familial inheritance has implications for future pregnancies and other family members, we should consider the value in performing a concurrent karyotype and contemplate a reduced or limited analysis of the chromosomes to identify balanced rearrangements. Finally, 1.4% of cases showed clinically significant CNAs elsewhere in the genome, unrelated to the apparent chromosomal rearrangement, demonstrating the power of microarray analysis to interrogate the entire genome.

## CONCLUSION

Microarray analysis has proven to be an important addition for the examination of the fetal chromosomes. Although the detection rates varied depending on the IFS, overall, 5.3% of nondemise cases demonstrated a clinically significant cytogenetic anomaly after microarray analysis. To the best of our knowledge, by removing cases with known abnormal karyotypes or family history of a known cytogenetic aberration, this detection rate represents an increase in detection of chromosome abnormalities in addition to that uncovered by routine cytogenetic testing. This detection rate is quite significant, especially if applied to the whole population of women undergoing invasive testing each year. Because the goal of invasive testing is to identify chromosome anomalies significant to fetal pathology, microarray testing should be considered a first-tier test for the diagnosis of cytogenetic aberrations in the fetus.

**WHAT'S ALREADY KNOWN ABOUT THIS TOPIC?**

1. Microarray testing has the ability to detect large and small, clinically significant copy number alterations.

**WHAT DOES THIS STUDY ADD?**

1. Through the analysis of a large prospective data set, detection rates of microarray for subsets of the pregnant population can be better understood.
2. This study provides strong support that microarray analysis should be seriously considered as a first-tier test in prenatal diagnostics.
